# Supplementary material for: Effects of fertilizations on soil bacteria and fungi communities in a degraded arid steppe revealed by high through-put sequencing
Source: PeerJ. 2018 Apr 16;6:e4623. doi: 10.7717/peerj.4623 (PMC5907784; doi:10.7717/peerj.4623)
Supplement: Table S1 — Different lowercase letters after the data within each soil layer represented significance at P < 0.05 (l.s.d). De, depth; Tr, treatment; CK, Control; P, 60 kg P ha−1; N, 100 kg N ha−1; NP, 60 kg P ha−1 plus 100 kg N ha−1; M, 4,000 kg sheep manure ha−1. Values were the means of three replicates ±SE. [file peerj-06-4623-s002.docx]

**Table. S1 Effects of fertilizations** **on bacterial phylum.** Different lowercase letters after the data within each soil layer represented significance at *P*<0.05 (l.s.d). De, depth; Tr, treatment. CK, Control; P, 60 kg P /ha; N, 100 kg N/ha; NP, 60 kg P /ha plus 100 kg N/ha; M, 4000 kg sheep manure /ha. Values were the means of three replicates ± SE.

| De | Tr | Actinobacteria | Proteobacteria | Acidobacteria | Chloroflexi | Gemmatimonadetes | Verrucomicrobia | Firmicutes | Bacteroidetes | Nitrospirae | Planctomycetes |
| --- | --- | --- | --- | --- | --- | --- | --- | --- | --- | --- | --- |
| 0-10 cm | CK | 8411.33±547.21a | 3926.33±304.52b | 3573.33±673.92a | 2035.67±42.43a | 805.00±91.83a | 546.67±90.12a | 274.33±2.73a | 511.67±71.21a | 256.67±7.13a | 165.00±17.01a |
|  | P | 8301.00±572.04a | 3576.67±244.70b | 3983.33±783.46a | 1726.00±96.59a | 766.67±38.20a | 747.00±196.06a | 626.00±139.52a | 462.67±54.33a | 223.33±4.67a | 178.00±40.61a |
|  | N | 9247.33±708.61a | 4179.0±427.85ab | 2972.33±831.63a | 1696.33±168.36a | 735.33±55.67a | 568.67±215.20a | 502.00±116.36a | 401.00±18.36a | 271.00±28.45a | 120.67±17.67a |
|  | NP | 8658.67±594.98a | 3754.67±314.22b | 3567.33±1095.50a | 1932.33±186.43a | 879.67±175.22a | 444.33±79.34a | 477.00±202.16a | 496.33±58.07a | 261.67±25.05a | 132.00±17.67a |
|  | M | 8041.67±147.72a | 5059.67±328.20a | 2712.00±328.94a | 2036.67±149.40a | 828.33±31.01a | 391.00±41.06a | 712.67±313.18a | 468.67±33.25a | 152.00±16.80b | 137.33±6.84a |
| 10-20 cm | CK | 8945.00±401.99a | 3379.33±436.31a | 3778.67±461.12a | 1578.33±164.49b | 940.00±80.75a | 832.33±251.35a | 344.67±52.41a | 246.33±62.21a | 328.33±53.96a | 219.33±34.71a |
|  | P | 9699.00±623.84a | 3728.00±144.76a | 2657.67±521.67a | 1404.00±41.86b | 915.33±110.04a | 698.67±115.79a | 512.00±80.80a | 328.00±11.59a | 336.67±28.05a | 188.67±7.62a |
|  | N | 9900.67±631.78a | 3722.33±248.27a | 2635.00±597.90a | 1500.00±126.50b | 877.00±85.59a | 742.33±269.75a | 351.33±88.77a | 283.67±15.07a | 382.67±35.30a | 213.67±42.88a |
|  | NP | 9012.67±257.25a | 3791.00±456.94a | 3150.67±415.84a | 1385.67±53.84b | 969.67±132.51a | 926.33±208.57a | 452.33±99.09a | 336.00±80.21a | 320.00±23.52a | 212.33±24.55a |
|  | M | 8360.33±676.43a | 3571.33±52.78a | 3936.00±366.80a | 2054.33±138.81a | 792.67±92.69a | 765.33±141.31a | 387.33±125.52a | 218.33±91.48a | 284.00±65.77a | 269.67±24.84a |
