# Supplementary material for: Effects of fertilizations on soil bacteria and fungi communities in a degraded arid steppe revealed by high through-put sequencing
Source: PeerJ. 2018 Apr 16;6:e4623. doi: 10.7717/peerj.4623 (PMC5907784; doi:10.7717/peerj.4623)
Supplement: Table S2 — Different lowercase letters after the data within each soil layer represented significance at P < 0.05 (l.s.d). De, depth; Tr, treatment; CK, Control; P, 60 kg P ha−1; N, 100 kg N ha−1; NP, 60 kg P ha−1 plus 100 kg N ha−1; M, 4,000 kg sheep manure ha−1. Values were the means of three replicates ±SE. “-” [file peerj-06-4623-s003.docx]

**Table S2 Effects of fertilizations** **on fungi phylum.** Different lowercase letters after the data within each soil layer represented significance at *P*<0.05 (l.s.d). De, depth; Tr, treatment. CK, Control; P, 60 kg P /ha; N, 100 kg N/ha; NP, 60 kg P /ha plus 100 kg N/ha; M, 4000 kg sheep manure /ha. Values were the means of three replicates ± SE. “-”

| De | Tr | Ascomycota | Basidiomycota | Glomeromycota | Zygomycota | Ciliophora | unclassified_  d_Eukaryota | Chytridiomycota | Arthropoda | unclassified_k__Fungi |
| --- | --- | --- | --- | --- | --- | --- | --- | --- | --- | --- |
| 0-10 cm | CK | 18583.33±639.98a | 2526.00±230.28a | 1671.33±484.78a | 698.33±69.80a | 621.67±95.10ab | 342.33±66.88a | 511.00±138.23a | 218.67±214.18a | 69.00±10.97a |
|  | P | 15365.33±1459.08a | 5053.67±1514.08a | 2313.67±160.63a | 686.00±181.51a | 353.00±118.20bc | 266.33±109.61a | 261.33±70.27a | 980.00±967.52a | 44.67±22.93a |
|  | N | 18507.67±634.84a | 2208.67±866.98a | 1523.67±366.28a | 1210.67±294.04a | 346.67±51.09bc | 342.00±156.86a | 178.33±6.12a | 564.67±562.17a | 78.00±10.97a |
|  | NP | 17342.00±1444.01a | 4693.00±1130.33a | 1367.00±135.65a | 642.00±115.94a | 264.67±85.08c | 517.67±132.64a | 331.00±40.51a | 40.33±39.34a | 71.00±8.62a |
|  | M | 18140.33±1398.30a | 3362.00±1202.26a | 1449.00±658.97a | 954.00±585.91a | 742.67±102.61a | 168.33±35.59a | 264.33±45.90a | 0.67±0.67a | 88.00±29.67a |
| 10-20 cm | CK | 19138.00±478.35a | 3422.00±880.39a | 566.00±163.40b | 1412.67±371.93a | 307.67±27.61a | 195.00±56.56a | 118.00±27.01b | 1.00±1.00a | 62.67±10.73a |
|  | P | 16528.67±2353.12a | 4931.67±2123.82a | 1480.67±477.70a | 1154.67±260.39a | 400.67±146.20a | 186.33±52.70a | 389.33±103.81a | 19.33±12.55a | 109.67±49.41a |
|  | N | 19266.67±1114.29a | 2798.67±864.82a | 501.33±108.55b | 1528.67±343.65a | 373.00±172.81a | 349.00±69.20a | 149.33±50.45b | 32.67±18.17a | 110.33±18.22a |
|  | NP | 19866.33±1565.68a | 2096.00±692.18a | 356.33±87.09b | 1580.33±739.83a | 366.67±81.87a | 312.67±70.60a | 440.33±64.12a | 18.00±18.00a | 90.33±20.33a |
|  | M | 18563.67±605.54a | 3257.33±599.77a | 655.33±233.74b | 1724.00±1101.27a | 303.00±28.04a | 177.00±19.50a | 192.00±24.13b | - | 107.33±39.56a |
