# Supplementary figures and images for: Effects of fertilizations on soil bacteria and fungi communities in a degraded arid steppe revealed by high through-put sequencing
Source: PeerJ. 2018 Apr 16;6:e4623. doi: 10.7717/peerj.4623 (PMC5907784; doi:10.7717/peerj.4623)

A

Microbial commiunity pieplot

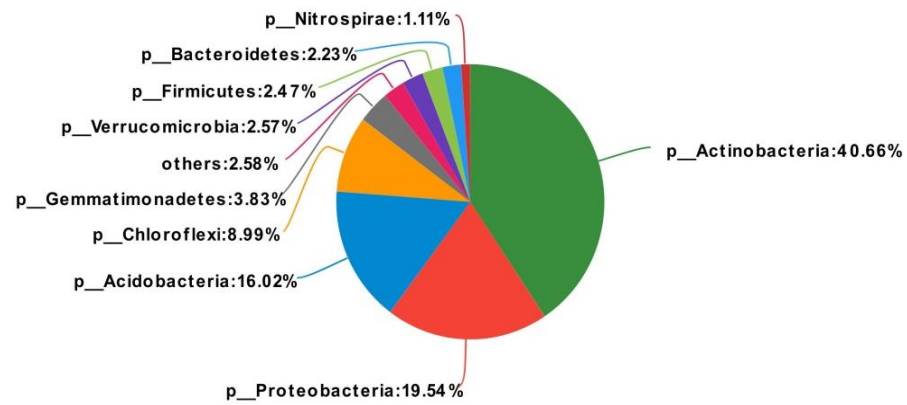

B

Microbial commiunity pieplot

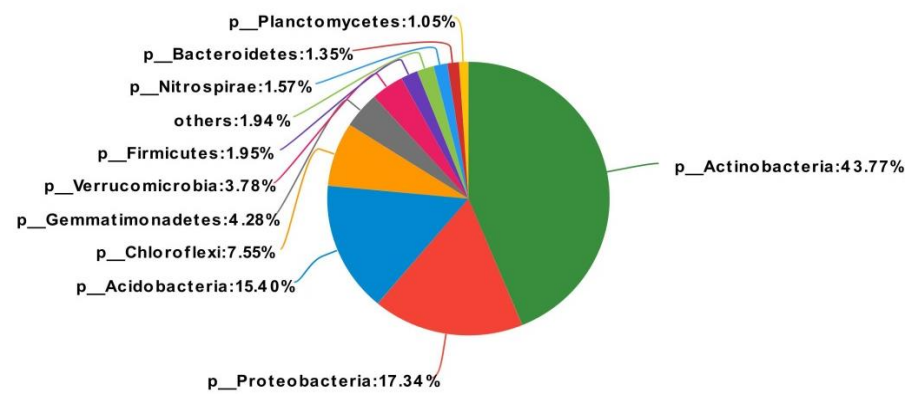

Supplement: Figure S1 — Averaged across all treatments. [file peerj-06-4623-s004.pdf]

A

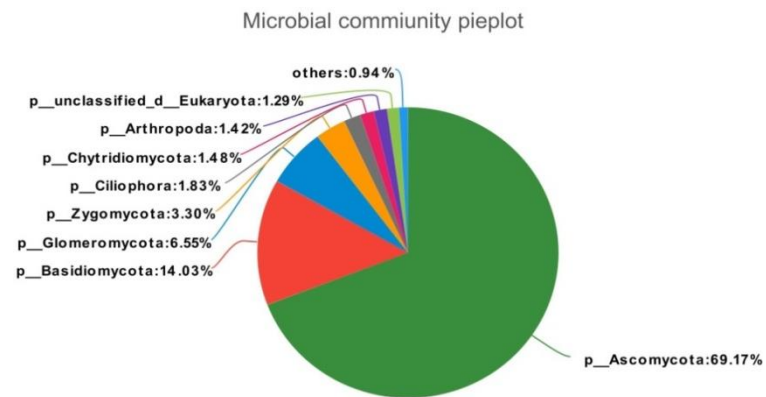

B

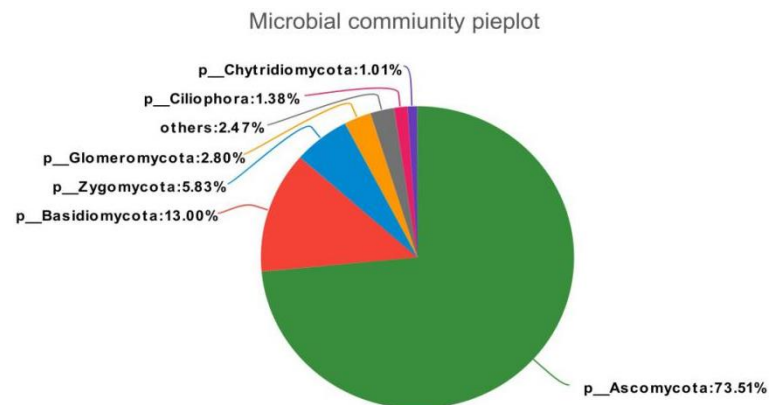

Supplement: Figure S2 — Averaged across all treatments. [file peerj-06-4623-s005.pdf]
